# Evidence of human-associated genetic similarity and a cryptic lineage in wild boar-derived *Ascaris* from Ishikawa Prefecture in Japan

**DOI:** 10.1186/s41182-025-00769-7

**Published:** 2025-07-01

**Authors:** Takahiro Matsumura, Kota Mochizuki, Kayoko Matsuo, Tomoyoshi Komiya, Masaharu Tokoro

**Affiliations:** 1 Faculty of Health and Medical Sciences, Hokuriku University, 1-1 Taiyogaoka, Kanazawa, Ishikawa 920-1180 Japan; 2 Department of Medical Zoology, Kanazawa Medical University, 1-1 Daigaku, Uchinada, Kahoku, Ishikawa 920-0293 Japan; 3 Faculty of Applied Biological Sciences, Gifu University, 1-1 Yanagido, Gifu, 501-1193 Japan; 4 Kumamoto Prefectural Aso Livestock Hygiene Office, Miyaji, Ichinomiya-Machi, Aso, Kumamoto 2639-1869-2612 Japan; 5 Department of Global Infectious Diseases, Graduate School of Medical Sciences, Kanazawa University, 13-1 Takara-Machi, Kanazawa, Ishikawa 920-0934 Japan

## Abstract

**Background:**

Two species of *Ascaris* nematodes infect humans: *Ascaris lumbricoides*, a human parasite, and *Ascaris suum*, which primarily infects pigs. Since these species are morphologically similar, molecular techniques are necessary for species identification in human *Ascaris* cases. A common method involves analyzing nucleotide sequences in the internal transcribed spacer 1 (ITS-1) region, particularly at positions 133 and 246. Although *Ascaris* nematodes have also been detected in wild boars, they are often classified as *A. suum* without molecular analysis due to their genetic similarity to pigs. In this study, we conducted molecular identification of *Ascaris* specimens collected from wild boars in Ishikawa Prefecture to examine their potential origin.

**Methods:**

Six *Ascaris* specimens from wild boars in Ishikawa Prefecture were analyzed by PCR and sequencing of the ITS-1 and COX1 regions. ITS-1 sequences were aligned to reference data, and phylogenetic analysis was performed using COX1 sequences.

**Results:**

Alignment analysis of the ITS-1 region revealed a nucleotide deletion at position 129, with guanine (G) at position 133 and thymine (T) at position 246. This sequence was 100% identical to the reference *A. lumbricoides* sequence derived from humans. However, phylogenetic analysis of the COX1 region revealed that these wild boar-derived genotypes belonged to a clade that has not been identified in human-derived *A. lumbricoides*.

**Discussion:**

The ITS-1 sequences of *Ascaris* from wild boars were identical to those of “*lumbricoides*” genotypes, suggesting possible past transmission from humans. However, COX1-based phylogenetic analysis revealed a distinct clade, indicating a potentially novel lineage within wild boars. These findings highlight the limitations of relying solely on ITS-1 for determining host origin and suggest that wild boars may serve as reservoirs of zoonotic *Ascaris* in Japan.

**Conclusions:**

This study highlights the presence of “*lumbricoides*” genotypes sequences in wild boars and their potential role in human ascariasis. Reevaluation of unexplained cases in relation to wild boar distribution is warranted, along with enhanced attention to zoonotic transmission risks.

## Background

Two species of *Ascaris* nematodes infect humans: *Ascaris lumbricoides*, a human parasite, and *Ascaris suum*, which primarily infects pigs (*Sus scrofa domesticus*). In Japan, improved sanitation and widespread screening programs have significantly reduced *A. lumbricoides* infections, with only sporadic cases currently reported [1]. However, in some instances, the transmission route remains unidentified, posing challenges for infection control [2, 3].

Meanwhile, *A. suum* has been detected in domestic pigs in Japan, and individuals at high risk of infection include pig farm workers, those handling pig-derived fertilizers, and their families [4]. Since the eggs and adult worms of *A. lumbricoides* and *A. suum* are morphologically indistinguishable, species identification in human *Ascaris* cases in Japan relies on nucleotide differences at positions 133 and 246 of the internal transcribed spacer 1 (ITS-1) region. This genetic marker has been used in endemic regions, such as Latin America and China, to differentiate human- and pig-derived *Ascaris* populations and confirm the absence of cross-infection [5–7].

Mitochondrial DNA analysis has also been employed for species identification. However, since *A. lumbricoides* and *A. suum* belong to the same lineage, mitochondrial DNA alone is insufficient for definitive classification [8, 9]. In contrast, phylogenetic analyses based on the COX1 region have delineated four major clades—A1, A2, B, and C. Clade A2 has been reported to predominantly comprise human-derived *Ascaris*, whereas clade C includes only pig-derived specimens, thereby enabling approximate inference of host origin [10]. Furthermore, that study reported that both “hybrid” and “*lumbricoides*” genotypes have also been identified among pig-derived *Ascaris*, suggesting potential complexity in host–parasite associations.

*Ascaris* nematodes have also been detected in wild boars (*Sus scrofa*). However, due to the close genetic relationship between wild boars and domestic pigs, these nematodes are often assumed to be *A. suum* without molecular identification in Japan [11]. This pattern is observed globally, as *Ascaris* from wild boars is typically classified as *A. suum* without genetic analysis [12, 13].

In this study, to investigate the origin of *Ascaris* nematodes in wild boars, we collected *Ascaris* samples from wild boars captured in Ishikawa Prefecture and conducted molecular analyses targeting the ITS-1 and cytochrome c oxidase subunit 1 (COX1) regions.

## Methods

Six adult *Ascaris* worms were collected between 2019 and 2024 from six wild boars captured in Ishikawa Prefecture, Japan. These wild boars were legally captured and euthanized as part of authorized wildlife control programs conducted in Ishikawa Prefecture. Their intestinal tracts were examined post-mortem, and adult *Ascaris* worms were manually collected from the small intestines. Genomic DNA was extracted from an approximately 5 mm × 5 mm piece of worm tissue using the Kaneka Easy DNA Extraction kit version 2 (Kaneka, Hyogo, Japan), following the manufacturer’s instructions. PCR was conducted using primer specific to ITS-1 and COX1 regions. The ITS-1 region was amplified with the primers F: 5′-CACATAAGTACTATTTGCGCG-3′ and R: 5′-CCACGAACCGAGTGATCCAC-3′ [14], while the COX1 region was amplified with the primers F: 5′-GCTCCTGATATGAGTTTTCCTCG-3′ and R: 5′-CTCAGACTGGTAACTATGAC-3′.

PCR amplification was performed using TakaRa PrimeSTAR polymerase under the following conditions: for the ITS-1 region, 35 cycles of denaturation at 98 °C for 10 s, annealing at 55 °C for 5 s, and extension at 72 °C for 30 s; for the COX1 region, 35 cycles of denaturation at 98 °C for 10 s, annealing at 56 °C for 5 s, and extension at 72 °C for 80 s.

PCR products were directly sequenced using the Sanger method (GENEWIZ, Inc., Tokyo, Japan). For the ITS-1 region, the same primers used for PCR were also used for sequencing. For the COX1 region, both the PCR primers and additional sequencing primers were used: F: 5′-TTTCTTTGGAACATATGAG-3′, F: 5′-GATATTATCTTGCATGATAC-3′, R: 5′-TATAAACCTCAGGATGACC-3′, and R: 5′-GCAAACACACTAATTATAGACC-3′. The obtained sequences were deposited in a DNA database. Sequence alignment was performed using IdentityX (https://home.hiroshima-u.ac.jp/~kei/IdentityX/index.html). The nucleotide sequences at positions 133 and 246 were compared with the reference sequence of *A. lumbricoides* and *A. suum* to determine species identity.

Additionally, phylogenetic analysis of the COX1 region was conducted using MEGA X with the Neighbor-Joining (NJ) method and Maximum-Likelihood (ML) method. Among the 70 reference sequences, those used by Cavallero et al. in their phylogenetic analysis were also included [10], allowing for comparison with established clades.

## Results

All DNA samples were successfully amplified, generating DNA fragments of 449 bp in length for ITS-1 and 1257 bp for COX1. Sanger sequencing yielded nucleotide sequences of 300 bp for the ITS-1 region (Accession Numbers: LC856584–LC856589) and 742 bp for the COX1 region (Accession Numbers: LC865723–LC865728). For the phylogenetic analysis of the COX1 region, 326 bp out of the 742-bp sequence was used to match the sequence length employed in the analysis by Cavallero et al. [10].

**Fig. 1:**
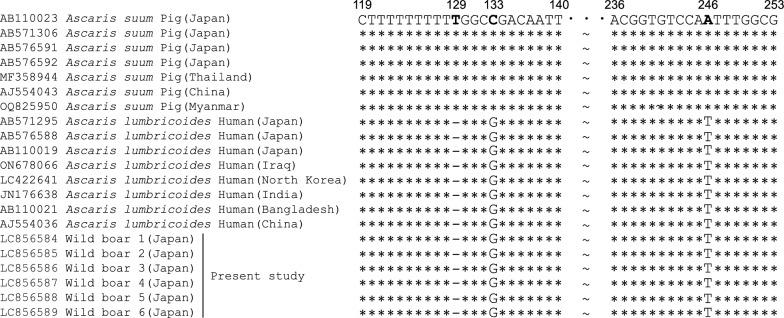
Comparative analysis of ITS-1 sequences. This figure presents a comparative analysis of ITS-1 sequences from *A. lumbricoides* (human origin), *A. suum* (pig origin), and *Ascaris* spp. obtained from wild boars in this study. Sequence similarities and differences are highlighted, with a particular focus on nucleotide variations at positions 129, 133, and 246

Alignment analysis of the ITS-1 region revealed a deletion at position 129, guanine (G) at position 133, and thymine (T) at position 246. These sequences showed 100% identical with the human-derived reference sequence of *A. lumbricoides* (Fig. 1), leading to their classification as “*lumbricoides*” genotypes.

**Fig. 2:**
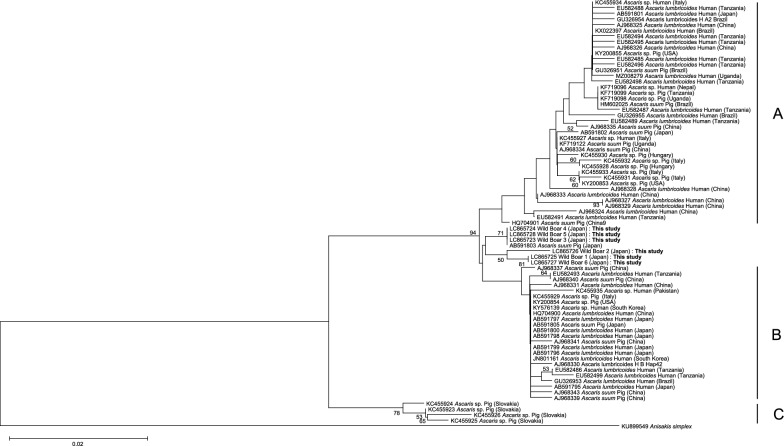
Phylogenetic tree of *Ascaris* sp. isolates from wild boars, constructed based on the COX1 gene using the Neighbor-Joining method. The phylogenetic tree was constructed using the Neighbor-Joining method with the Tamura–Nei model. *Anisakis simplex* was included as an outgroup. The total sequence length was 326 bp. Bootstrap support values (≥ 50%) are shown at the nodes and were calculated using 1000 replicates. The reference clades A, B, and C displayed in the phylogenetic trees were incorporated based on the classification proposed by Cavallero et al. [10]

**Fig. 3:**
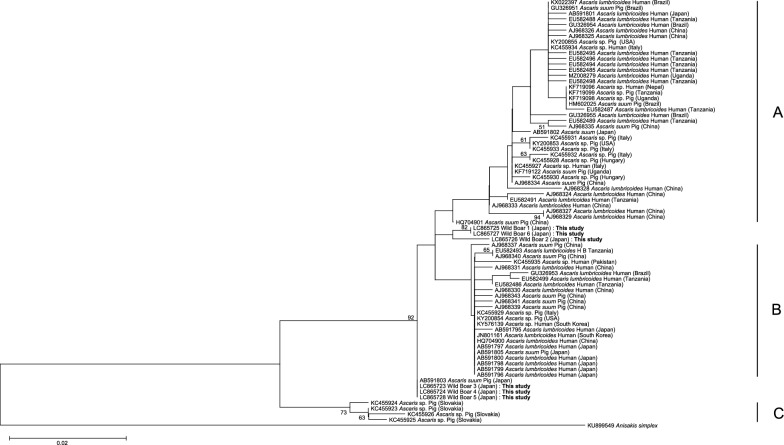
Phylogenetic tree of *Ascaris* sp. isolates from wild boars, constructed based on the COX1 gene using the Maximum-Likelihood method. The phylogenetic tree was constructed using the Maximum-Likelihood method with the Tamura–Nei model. *Anisakis simplex* was included as an outgroup. The total sequence length was 326 bp. Bootstrap support values (≥ 50%) are shown at the nodes and were calculated using 1000 replicates. The reference clades A, B, and C displayed in the phylogenetic trees were incorporated based on the classification proposed by Cavallero et al. [10]

Phylogenetic analysis based on the COX1 region revealed that the wild boar-derived *Ascaris* specimens did not cluster within the human-derived *A. lumbricoides* clade or any of the globally recognized clades reported to date (Figs. 2, 3). The reference clades A, B, and C displayed in the phylogenetic trees were incorporated based on the classification proposed by Cavallero et al. [10].

In the Neighbor-Joining (NJ) tree, the wild boar samples formed a novel cluster situated between clades A and B. In the Maximum Likelihood (ML) tree, wild boars 1, 2, and 6 also formed a distinct cluster between clades A and B, while wild boars 3, 4, and 5 grouped separately between clades B and C, alongside previously reported pig-derived *Ascaris* isolates from Japan.

## Discussion

In Japan, *Ascaris* nematodes from wild boars have long been regarded as *A. suum* [11]. However, molecular analysis in this study revealed that the ITS-1 region exhibited a human-derived genetic type, while the COX1 region did not correspond to any known species, instead suggesting the presence of a novel clade.

Regarding the introduction of *Sus scrofa* history in Japan, modern pig breeds, such as Landrace, Hampshire, and Duroc, originating from Europe and North America, have been predominant since the twentieth century [15]. In contrast, native Japanese wild boars are believed to have been introduced from northeastern China multiple times during the Pleistocene [16]. The ITS-1 results suggest that “*lumbricoides*” genotypes may have established a population in wild boars through historical human–boar interactions. This possibility is further supported by previous reports of “*lumbricoides*” genotypes being detected in pigs, suggesting that wild boars could have become infected through historical interactions between humans and wild boars [10]. Additionally, a report by Loreille et al. documented *Ascaris* eggs of human origin dating back approximately 30,000 years, prior to pig domestication, from archeological site remains in France [17]⁠. In the United States, Goncalves et al. confirmed the presence of *Ascaris* eggs in human feces from a period before the introduction of pigs [18]. These findings support the possibility that transmission occurred from humans to wild boars.

The COX1 findings further imply that these nematodes may have formed a distinct genetic lineage within the wild boar population. Although this study is based on a limited sample size, it highlights the need for caution when inferring host origin in Japanese *A. lumbricoides* cases solely based on ITS-1 analysis.

Molecular classification using the COX1 region may provide a broader but useful indication of whether *Ascaris* samples originate from wild boars or humans. In Japan, sporadic domestic *A. lumbricoides* infections have been linked to organic vegetables fertilized with human feces, soil-contaminated imported vegetables, and imported kimchi [19]. However, pinpointing the exact source of infection remains difficult, as many cases lack a clear transmission route, complicating efforts to implement effective preventive measures. While previous studies have primarily focused on known transmission pathways, our findings suggest that contamination by wild boar feces should be considered a potential source of infection.

A better understanding of transmission routes is crucial for developing more effective prevention strategies. In recent years, wild boars have increasingly encroached on human settlements and caused agricultural damage, bringing them into closer contact with humans. Consequently, their potential role as an infection source warrants further attention [20]. Additionally, international studies have reported an increase in protozoan infections following floods [21], and flood-affected populations have been found to be at higher risk of ascariasis [22]. In September 2024, heavy rainfall disasters occurred in the Noto region of Ishikawa Prefecture. Given that wild boars in this region harbor “*lumbricoides*” genotypes, the potential for an increase in ascariasis cases following such disasters should not be overlooked. In addition to *Ascaris*, post-flood conditions may also elevate the risk of infection through food contamination with mud and debris. Therefore, maintaining proper hygiene practices and disseminating risk-related information are essential for infection prevention.

However, as this study focused solely on wild boars in Ishikawa Prefecture, further genetic analyses of *Ascaris* nematodes from wild boars in other regions are necessary to better understand the nationwide distribution of *Ascaris* species in Japan.

## Conclusions

This study identified “*lumbricoides*” genotypes for ITS-1 region in wild boars from Ishikawa Prefecture, suggesting that wild boars may serve as a potential reservoir for zoonotic *Ascaris* in Japan. Furthermore, phylogenetic analysis based on the COX1 region revealed a distinct clade, indicating that a cryptic lineage of *Ascaris* may exist in wild boar populations. These findings highlight the need for further investigation to clarify this cryptic genetic diversity and to better understand its potential public health implications.

## Data Availability

No datasets were generated or analyzed during the current study.
